# Epidemic Characteristics of HIV Drug Resistance in Hefei, Anhui Province

**DOI:** 10.3390/pathogens11080866

**Published:** 2022-07-31

**Authors:** Shan Zheng, Jianjun Wu, Jingjing Hao, Dong Wang, Zhongwang Hu, Lei Liu, Chang Song, Jing Hu, Yanhua Lei, Hai Wang, Lingjie Liao, Yi Feng, Yiming Shao, Yuhua Ruan, Hui Xing

**Affiliations:** 1 State Key Laboratory of Infectious Disease Prevention and Control, National Center for AIDS/STD Control and Prevention, Chinese Center for Disease Control and Prevention, Beijing 102206, China; zhengshan0926@163.com (S.Z.); hao666jingjing123@163.com (J.H.); wangdong19971229@163.com (D.W.); cdcliulei@163.com (L.L.); songchang604@163.com (C.S.); angelahu46@sina.com (J.H.); liaolj@chinaaids.cn (L.L.); fengyi@chinaaids.cn (Y.F.); yshao@bjmu.edu.cn (Y.S.); ruanyuhua92@163.com (Y.R.); 2 Anhui Provincial Center for Disease Control and Prevention, Hefei 230601, China; wujianjun823@126.com; 3 Hefei Center for Disease Control and Prevention, Hefei 230061, China; hzw1100@126.com (Z.H.); 13839084827@163.com (Y.L.); swan_qi@163.com (H.W.)

**Keywords:** HIV, drug resistance, molecular network, transmission

## Abstract

To study the characteristics of HIV pretreatment drug resistance (PDR) and acquired drug resistance (ADR) in Hefei, a cross-sectional survey was used to collect 816 samples from newly reported HIV infections from 2017 to 2020 and 127 samples from HIV infections with virological failure from 2018 to 2019 in Hefei. HIV drug resistance levels and drug resistance mutations were interpreted using the Stanford Drug Resistance Database. Molecular networks were constructed by HIV-TRACE. Among the newly reported infections in Hefei, the prevalence of PDR was 6.4% (52/816). The drug resistance mutations were mainly V179E/D/T (12.4%), K103N (1.3%), and V106I/M (1.3%). In addition, it was found that the CRF55_01B subtype had a higher drug resistance rate than other subtypes (*p* < 0.05). Molecular network analysis found that K103N and V179E may be transmitted in the cluster of the CRF55_01B subtype. The prevalence of ADR among HIV infections with virological failure was 38.6% (49/127), and the drug resistance mutations were mainly M184V (24.4%), K103N/S (15.7%), Y181C (11.0%), G190S/A/E (10.2%), and V106M/I (10.2%). The molecular network was constructed by combining HIV infections with virological failure and newly reported infections; M184V and Y181C may be transmitted between them. The chi-square trend test results indicated that the higher the viral load level, the greater the number of newly reported infections linked to the infections with virological failure in the molecular network. In conclusion, interventions should focus on infections of the CRF55_01B subtype to reduce the transmission of drug-resistant strains. However, improving the treatment effect of HIV infections is beneficial for reducing the second-generation transmission of HIV.

## 1. Introduction

Antiretroviral therapy (ART) can effectively reduce the mortality rate and transmission of HIV. By 2020, there were about 37.7 million infections worldwide, the treatment coverage rate reached 73%, and the success rate of treatment was 70–98% [1]. However, there are still many patients for whom ART fails, and some of them may die. HIV drug resistance is one of the important reasons for the failure of ART. Pretreatment drug resistance and acquired drug resistance during ART are key factors to consider when choosing treatment options. The molecular network has been widely used to study the characteristics of HIV transmission in recent years. Many studies have combined drug resistance and the molecular network to study the transmission of drug-resistant strains [2,3], and some studies have found that antiviral therapy has a great effect on reducing the second-generation transmission of HIV by using molecular network analysis [4].

The epidemic of HIV in Hefei originated from the early illegal blood collection and supply events in northern Anhui province. The first AIDS case was reported in 1997. After 2004, the main route of HIV infection gradually changed to heterosexual (HET) transmission, and after 2013, the transmission among men who have sex with men (MSM) increased rapidly [5,6]. There has been no report on HIV prevalence in Hefei in recent years. This study analyzed the drug resistance and transmission of newly reported infections from 2017 to 2020 and the infections with virological failure from 2018 to 2019 in Hefei to provide data support for the development of individualized precision therapy. At the same time, by analyzing the molecular network links between the infections with virological failure and newly reported infections, we hope to understand the impact of antiviral therapy on HIV transmission in Hefei.

## 2. Results

### 2.1. Characteristics of the Study Population

A total of 816 sequences from newly reported HIV infections and 127 sequences from HIV infections with virological failure were available. Among the newly reported HIV infections, 39.0% (318/816) were 18–29 years old, 76.8% (627/816) were male, 61.9% (505/816) were MSM, 46.4% (379/816) were single, 56.6% (462/816) had obtained a high school and above education, and 67.4% (550/816) had a CD4^+^ T cell count of <500 cells/µL (Table 1). The main subtypes were CRF07_BC (41.4%), CRF01_AE (38.1%), and CRF55_01B (6.3%), and the main subclusters were CRF07_BC-N (34.9%), CRF01_AE-cluster 4 (24.4%), and CRF01_AE-cluster 5 (8.1%). Among the infections with virological failure, 60.6% (77/127) were 18–39 years old, 85.8% (109/127) were male, 56.7% (72/127) were MSM, and 45.7% (58/127) had a viral load of 1000–10,000 copies/mL. The antiviral treatment regimens used were mainly EFV/LPV/r/NVP+3TC+AZT/TDF (Table 2).

### 2.2. Drug Resistance Analysis

The PDR frequency of newly reported HIV infections was 6.4% (52/816). Drug resistance to protease inhibitors (PIs), nucleoside reverse transcriptase inhibitors (NRTIs), and non-nucleoside reverse transcriptase inhibitors (NNRTIs) accounted for 2.0%, 1.2%, and 3.8%, respectively. The main drug resistance mutations were V179E/D/T (12.4%), K103N (1.3%), and V106I/M (1.3%) (Table 3). Of the 101 infections carrying V179E/D/T, 90 cases carrying V179E/D/T alone were not resistant to NNRTIs. Resistance occurred when V179E/D was combined with E138A/G or V106I or K103N (Table 4). With drug resistance as the dependent variable and age, sex, ethnicity, education, marital status, route of HIV infection, subtype, CD4 count, and sampling time as independent variables, univariate and multivariate logistic regression analysis showed that, after adjusting for other factors, the comparison of subtype was statistically significant (*p* < 0.05). The drug resistance rate of CRF55_01B was higher than that of other subtypes (Table 5).

The ADR frequency of HIV infections with virological failure was 38.6% (49/127), and drug resistance to PIs, NRTIs, and NNRTIs accounted for 2.4%, 29.1%, and 36.2%, respectively. The main drug resistance mutations were M184V (24.4%), K103N/S (15.7%), Y181C (11.0%), G190S/A/E (10.2%), and V106M/I (10.2%) (Table 6).

### 2.3. HIV Molecular Network Analysis

**Figure 1:**
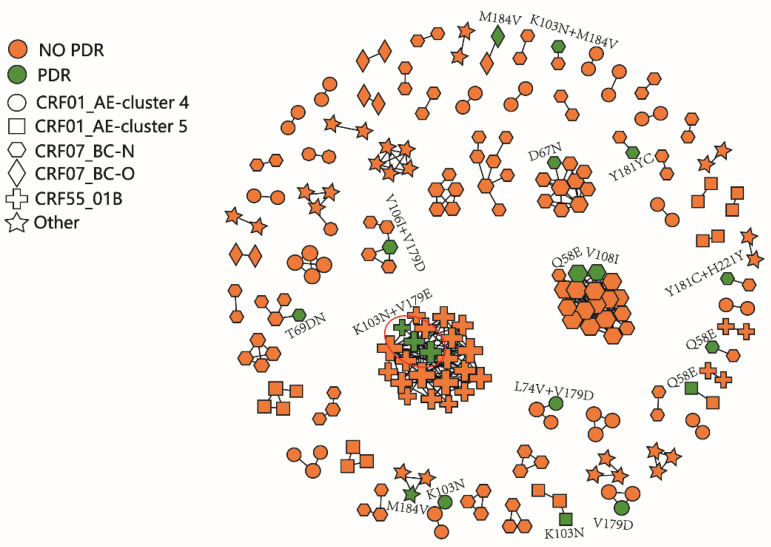
The molecular network of newly reported infections in Hefei. The green color represents infections with drug resistance mutations. Different shapes represent different subtypes. The red circles show infections with the same resistance mutations.

The molecular network of newly reported infections was constructed under the threshold of 0.5% gene distance; in total, 27.1% (221/816) of the sequences from 69 clusters were enrolled in the molecular network. There were 19 infections with drug-resistant mutations in the network. Three infections with K103N and V179E appeared in the transmission cluster of the CRF55_01B subtype, which was also the largest transmission cluster. The molecular network diagram is shown in Figure 1.

**Figure 2:**
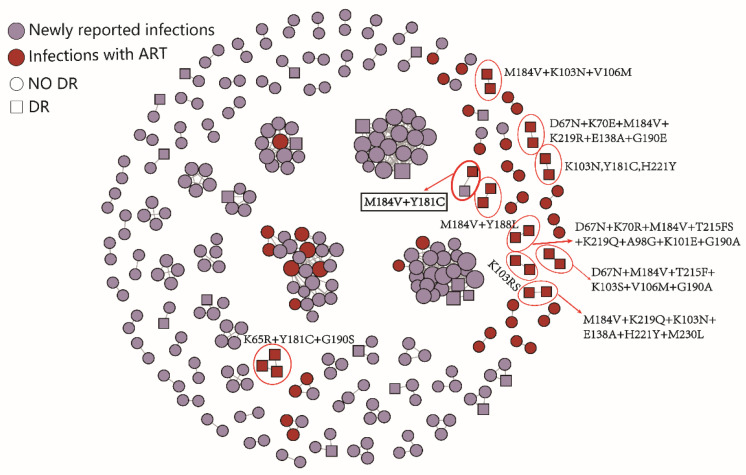
The molecular network of HIV infections with virological failure and newly reported infections in Hefei. The red circles are clusters associated with ART failures with resistance mutations.

In order to analyze the impact of ART on HIV transmission in Hefei, the molecular network was constructed by combining HIV infections with virological failure and newly reported infections. In total, 93 clusters were formed, including 26 drug-resistant transmission clusters. In the network, 21 infections with virological failure were directly or indirectly transmitted with 39 newly reported infections. There were 10 clusters associated with the failure of ART with resistance mutations, most of which were small clusters containing two nodes. There was also one infection with virological failure that was connected with one newly reported infection and carried M184V and Y181C. The molecular network diagram is shown in Figure 2.

The infections with virological failure were divided into three groups according to the level of viral load, and the number of links between infections with virological failure and newly reported infections was compared. The chi-square trend test results were statistically significant (*Z* = −2.98, *p* = 0.001), indicating that the higher the viral load level, the greater the number of newly reported infections linked to the infections with virological failure in the molecular network (Table 7).

## 3. Discussion

This study shows that the HIV epidemic population in Hefei is mainly composed of MSM. Among the newly reported HIV/AIDS cases in China in 2021, 71.4% were sexually transmitted, and homosexual transmission accounted for 26.5%. National surveillance data show that MSM are at high risk of HIV infection. MSM are disproportionally affected by HIV infection. Thus, it is necessary to design targeted interventions aimed at facilitating prevention, including through access to pre-exposure and post-exposure prophylaxis. At the same time, interventions should be made to reduce the stigma of MSM, which may improve the detection rate and HIV prevention and treatment services among MSM [7,8].

There are various HIV subtypes in Hefei, but CRF07_BC and CRF01_AE are the main subtypes of HIV infection in China. In addition, some new recombinant strains are also prevalent. As a strain originating from MSM, CRF55_01B has increased rapidly in recent years. The CRF55_01B strain was found in all provinces of China and has been transmitted from MSM to HET [9]. Attention should also be paid to the prevalence of new recombinant strains in the local area, and timely interventions should be made to prevent their large-scale transmission.

The PDR frequency of newly reported infections in Hefei was 6.4%, higher than previous investigation results on the drug resistance rate of HIV infections before treatment in Hefei (4.6%) [10]. In 2018, 12 drugs recommended by the WHO were selected to determine drug resistance in the National HIV molecular epidemiology survey, with an average drug resistance rate of 4.4% [11]. The overall PDR in China is at a low level. However, the PDR is already at moderate levels in some cities, such as Yunnan (7.5%) [12] and Tianjin (11.5%) [13]. The newly reported HIV infections have the highest drug resistance rate for non-nucleoside drugs, mainly for NVP and EFV, and the drug-resistant mutations are V179E/D/T, K103N, and V106I/M. This is related to the widespread use of non-nucleoside drugs and the strong spread and adaptability of these drug-resistant mutations, which are easy to detect due to their persistence in the body [2]. In this study, most of the patients carrying V179E/D/T alone did not develop drug resistance, which may be due to naturally polymorphic mutations. The high prevalence of V179 may be, in part, driven by natural V179 polymorphisms.

There were differences in the drug resistance rates among different subtypes in Hefei. Although CRF55_01B was not the most infectious, its drug resistance rate was higher than that of the other subtypes, which was consistent with the results of the national HIV molecular epidemic survey in China in 2018. Among the clusters containing drug-resistant mutations identified by the molecular network, the infections with both K103N and V179E gathered in the clusters of CRF55_01B. Previous studies have shown that V179E is a signature mutation in the CRF55_01B strain, but when it is combined with other drug-resistant mutations, it shows drug resistance [14]. Further research is needed on the mechanism of drug resistance of V179E and the impact of ART.

The ADR frequency of HIV infections with virological failure in Hefei was 38.6%, lower than that in China (44.7%) [15]. Compared with the newly reported infections, M184V, Y181C, and G190S/A/E were added to the main resistance mutations. The transmission of M184V and Y181C occurred between the infection with virological failure and the newly reported infection. Studies have shown that M184V easily mutates into the wild strain in the host in a short time, and its replication capacity is enhanced [16], indicating that the transmission between infections may occur quickly. The failure of ART with a high viral load has more newly reported infections connected in the molecular network and may be more transmissible, thus the antiviral treatment for the viral load reduction can reduce the transmission of HIV. Therefore, the effect of antiviral treatment should be improved in order to effectively reduce the second generation of transmission.

In conclusion, the HIV epidemic population in Hefei city is mainly MSM, and the main subtypes are CRF07_BC, CRF01_AE, and CRF55_01B. Whether K103N and V179E actually spread easily in CRF55_01B requires further research. Drug resistance is one of the major issues affecting the effects of antiviral therapy. Countries should take active actions and cooperate extensively to strengthen HIV drug resistance monitoring, adjust treatment plans according to specific conditions, improve drug compliance, speed up research and development and the use of new drugs, improve treatment effectiveness, and strive to achieve the goal of ending the AIDS epidemic at an early date. This study had some limitations. The lack of basic information of some research objects may have some impact on the analysis of the influencing factors.

## 4. Materials and Methods

### 4.1. Study Population and Design

Newly reported HIV infections from 2017 to 2020 and HIV infections with virological failure from 2018 to 2019 in Hefei were collected by sampling. The inclusion criteria of the newly reported HIV infections were as follows: age ≥ 18 years; patients with HIV infection who had not received any antiviral treatment; and patients who filled out questionnaires and signed informed consent forms. The inclusion criteria for HIV infections with virological failure were as follows: age ≥ 18 years; at least 6 months of antiviral therapy by the time of sampling; virological failure (plasma samples from patients under treatment in Hefei were quantified for their viral load twice per year from 2018 to 2019. ADR was tested immediately for patients with a viral load greater than 1000 copies/mL); and patients who filled out questionnaires and signed informed consent forms.

### 4.2. Laboratory Tests

Viral RNA was extracted from each plasma sample using the QIAamp viral RNA mini kit (Qiagen, Hilton, Germany). A nested polymerase chain reaction (PCR) was used to amplify the HIV *pol* gene fragments (HXB2: 2253–3553nt) by an in-house method [17], covering the full-length protease (amino acids 1–99) and the first 240 amino acids of reverse transcriptase codons. Sequences were obtained by Sanger sequencing.

### 4.3. Subtype and Drug Resistance Analysis

Sequences were spliced using Sequencher 4.10.1 (GeneCodes Corporation, Ann Arbor, MI, USA) and aligned using Mafft 7.037. FastTree and IQ-Tree were used to build the phylogenetic tree. Clusters with a bootstrap value higher than 90% were judged as the same subtype. Sequences were uploaded to the Stanford Drug Resistance Database (Stanford HIVDB, https://hivdb.stanford.edu accessed on 15 September 2021 8.9-1 version) to obtain the results of the listed 20 kinds of drug resistance score and degree: 15–29 was classified as low resistance (L), 30–59 was classified as moderate resistance (I), and 60 or above was classified as high resistance (H). In this study, resistance was defined as a resistance score of 15 or greater for at least one drug [18], and the corresponding drug-resistant mutations were obtained.

### 4.4. HIV Molecular Network Construction

The molecular network was constructed using HIV-TRACE [19]. Aligned *pol* sequences were used to calculate pairwise genetic distances using the Tamura-Nei 93 model [20]. All sequences were longer than 1000 bp, and the ambiguous nucleotides were less than 5%. According to the molecular network guidelines recommended by the CDC in the USA and the CDC in China, a genetic distance threshold of 0.5% substitutions per site was selected to identify transmission relationships over two to three years in Hefei [21]. The results and subsequent molecular network diagram can be found on the following webpage: https://veg.github.io/hivtrace-viz/ accessed on 20 September 2021.

### 4.5. Statistical Analysis

Statistical analyses were conducted in SAS 9.4 (SAS Institute Inc., Cary, NC, USA). Univariate and multivariate logistic regression models were used to analyze the influencing factors of PDR. The chi-square trend test was used for comparison of the number of newly reported infections linked to the failure of ART in different viral load groups.

## Figures and Tables

**Table 1:** Demographic characteristics of newly reported HIV infections in Hefei from 2017 to 2020.

Variable	Number	%
Total	816	100.0
Age (years)		
18–29	318	39.0
30–39	147	18.0
40–49	100	12.3
≥50	121	14.8
Unknown	130	15.9
Sex		
Male	627	76.8
Female	59	7.2
Unknown	130	15.9
Ethnicity		
Han	680	83.3
Other	6	0.7
Unknown	130	15.9
Education		
Illiterate	81	9.9
Primary or junior high school	143	17.5
Senior high school or higher	462	56.6
Unknown	130	15.9
Marital status		
Single	379	46.4
Married	210	25.7
Divorced/widowed	97	11.9
Unknown	130	15.9
Route of infection *		
MSM	505	61.9
HET	178	21.8
IDU	3	0.4
Unknown	130	15.9
Subtype		
CRF55_01B	51	6.3
CRF01_AE-cluster 4	199	24.4
CRF01_AE-cluster 5	66	8.1
CRF07_BC-N	285	34.9
Other	215	26.3
CD4 count before ART (cells/µL)		
<200	214	26.2
200–350	196	24.0
350–500	140	17.2
>500	136	16.7
Unknown	130	15.9
Sampling time		
2017	66	8.1
2018	162	19.9
2019	215	26.3
2020	373	45.7

* MSM, men who have sex with men; HET, heterosexual; IDU, injecting drug users.

**Table 2:** Demographic characteristics of HIV infections with virological failure in Hefei from 2018 to 2019.

Variable	Number	%
Total	127	100.0
Age (years)		
18–29	24	18.9
30–39	53	41.7
40–49	15	11.8
≥50	33	26.0
Unknown	2	1.6
Sex		
Male	109	85.8
Female	16	12.6
Unknown	2	1.6
Ethnicity		
Han	126	99.2
Other	1	0.8
Marital status		
Single	75	59.1
Married	35	27.6
Divorced/widowed	15	11.7
Unknown	2	1.6
Route of infection *		
MSM	72	56.7
HET	49	38.6
IDU	1	0.8
Unknown	5	3.9
VL (copies/mL)		
1000–10,000	58	45.7
10,000–50,000	41	32.3
>50,000	28	22.0
ART regimen *		
EFV+3TC+TDF	47	37.0
EFV+3TC+AZT	16	12.6
LPV/r+3TC+TDF	24	18.9
LPV/r+3TC+AZT	19	15.0
NVP+3TC+AZT	12	9.4
Other	9	7.1
Sampling time		
2018	59	46.5
2019	68	53.5

* MSM, men who have sex with men; HET, heterosexual; IDU, injecting drug users. EFV, efavirenz; 3TC, lamivudine; TDF, tenofovir disoproxil; AZT, zidovudine; LPV/r, lopinavir/ritonavir; NVP, nevirapine.

**Table 3:** Drug resistance and mutations of newly reported HIV infections in Hefei from 2017 to 2020.

Antiretroviral Drug	Number	%	HIV Drug Resistance Mutations (n, %)
Total	52	6.4	
PIs	16	2.0	
ATV	1	0.1	D30N (1, 0.1), K20T (2, 0.2), L10F (1, 0.1), L90M (1, 0.1), M46I (7, 0.9), Q58E (7, 0.9)
FPV	2	0.2	
IDV	1	0.1	
LPV/r	1	0.1	
NFV	9	1.1	
SQV	1	0.1	
TPV	7	0.9	
NRTIs	10	1.2	
ABC	8	1.0	A62V (2, 0.2), D67N (2, 0.2), K219E/R (3, 0.4), K65R (1, 0.1), K70T (1, 0.1), L74L/V (2, 0.2), M184V (4, 0.5), T215I (1, 0.1), T69DN (1, 0.1)
AZT	2	0.2	
D4T	4	0.5	
DDI	6	0.7	
FTC	5	0.6	
3TC	5	0.6	
TDF	2	0.2	
NNRTIs	31	3.8	
DOR	8	1.0	V179E/D/T (101, 12.4), K103N (11, 1.3), V106I/M (11, 1.3), V108I (4, 0.5), E138G/A/Q (5, 0.6), Y181H/C (3, 0.3), G190S (1, 0.1), K101E (1, 0.1), P225H (1, 0.1), F227L (1, 0.1), H221Y (1, 0.1)
EFV	20	2.5	
ETR	8	1.0	
NVP	25	3.1	
RPV	13	1.6	

PIs, protease inhibitors; NRTIs, nucleoside reverse transcriptase inhibitors; NNRTIs, non-nucleoside reverse transcriptase inhibitors. ATV, atazanavir; FPV, fosamprenavir; IDV, indinavir; LPV/r, lopinavir/ritonavir; NFV, nelfinavir; SQV, saquinavir; TPV, tipranavir; ABC, abacavir; AZT, zidovudine; D4T, stavudine; DDI, didanosine; FTC, emtricitabine; 3TC, lamivudine; TDF, tenofovir disoproxil; DOR, dorivirine; EFV, efavirenz; ETR, etravirine; NVP, nevirapine; RPV, rilpivirine.

**Table 4:** Drug resistance to NNRTIs in newly reported HIV infections with V179D/E/T.

HIV Drug Resistance Mutations	Number (%)	Level of Drug Resistance *	NNRTIs
Total	101		
V179D/E/T	90 (89.1)	-	-
V179D	1 (1.0)	I	EFV, NVP
	1 (1.0)	L	RPV
E138A, V179E	1 (1.0)	L	ETR, RPV
E138G, V179E	3 (3.0)	L	EFV, ETR, NVP, RPV
V106I, V179D	1 (1.0)	L	ETR, NVP, RPV
K103N, V179E	4 (4.0)	H	EFV, NVP

* L, low level; I, intermediate level; H, high level. NNRTIs, non-nucleoside reverse transcriptase inhibitors. EFV, efavirenz; ETR, etravirine; NVP, nevirapine; RPV, rilpivirine.

**Table 5:** Factors associated with pretreatment HIVDR among newly reported HIV infections.

Variable	Number	Drug Resistance, n (%)	OR * (95% *CI*)	*p*	AOR * (95% *CI*)	*p*
Total	816	52 (6.4)				
Age (years)						
18–29	318	17 (5.3)	1.00			
30–39	147	9 (6.1)	1.16 (0.50–0.66)	0.735		
40–49	100	6 (6.0)	1.13 (0.43–2.95)	0.803		
≥50	121	12 (9.9)	1.95 (0.90–4.21)	0.090		
Unknown	130	8 (6.2)	1.16 (0.49–2.76)	0.736		
Sex						
Male	627	39 (6.2)	1.00			
Female	59	5 (8.5)	1.40 (0.53–3.69)	0.501		
Unknown	130	8 (6.2)	0.99 (0.45–2.17)	0.977		
Ethnicity						
Han	680	44 (6.5)	1.00			
Other	6	0 (0.0)	-	-		
Unknown	130	8 (6.2)	0.95 (0.44–2.06)	0.893		
Education						
Illiterate	81	4 (4.9)	1.00			
Primary or junior high school	143	15 (10.5)	2.26 (0.72–7.04)	0.161		
Senior high school or higher	462	25 (5.4)	1.10 (0.37–3.25)	0.861		
Unknown	130	8 (6.2)	1.26 (0.37–4.33)	0.711		
Marital status						
Single	379	20 (5.3)	1.00			
Married	210	14 (6.7)	1.28 (0.63–2.59)	0.490		
Divorced/widowed	97	10 (10.3)	2.06 (0.93–4.57)	0.074		
Unknown	130	8 (6.2)	1.18 (0.51–2.74)	0.705		
Route of infection *						
MSM	505	29 (5.7)	1.00			
HET	178	15 (8.4)	1.51 (0.79–2.89)	0.212		
IDU	3	0 (0.0)	-	-		
Unknown	130	8 (6.1)	1.08 (0.48–2.41)	0.858		
Subtype						
CRF55_01B	51	6 (11.8)	1.00		1.00	
CRF01_AE-cluster 4	199	8 (4.0)	0.31 (0.10–0.95)	0.040	0.26 (0.08–0.81)	0.021
CRF01_AE-cluster 5	66	3 (4.5)	0.36 (0.09–1.50)	0.160	0.33 (0.07–1.42)	0.036
CRF07_BC-N	285	15 (5.2)	0.42 (0.15–1.13)	0.086	0.35 (0.12–0.98)	0.045
Other	215	20 (9.3)	0.77 (0.29–2.03)	0.595	0.60 (0.21–1.70)	0.334
CD4 count before ART (cells/µL)						
<200	214	15 (7.0)	1.00			
200–350	196	13 (6.6)	0.94 (0.44–2.03)	0.880		
350–500	140	7 (5.0)	0.70 (0.28–1.76)	0.446		
>500	136	9 (6.6)	0.94 (0.40–2.21)	0.888		
Unknown	130	8 (6.2)	0.87 (0.36–2.11)	0.758		
Sampling time						
2017	66	4 (6.1)	1.00			
2018	162	8 (4.9)	0.81 (0.23–2.77)	0.731		
2019	215	10 (4.7)	0.76 (0.23–2.50)	0.646		
2020	373	30 (8.0)	1.36 (0.46–3.98)	0.580		

* OR, odds ratio. AOR, adjusted odds ratio. MSM, men who have sex with men; HET, heterosexual; IDU, injecting drug users.

**Table 6:** Drug resistance and mutations of HIV infections with virological failure in Hefei from 2018 to 2019.

Antiretroviral Drug	Number	%	HIV Drug Resistance Mutations (n, %)
Total	49	38.6	
PIs	3	2.4	
ATV	2	1.6	M46I (2, 1.6), I54V (2, 1.6), L76V (1, 0.8), V82A (2, 1.6), L10F (2, 1.6)
DRV	1	0.8	
FPV	2	1.6	
IDV	2	1.6	
LPV/r	2	1.6	
NFV	2	1.6	
SQV	2	1.6	
TPV	3	2.4	
NRTIs	37	29.1	
ABC	36	28.3	D67N/T (9, 7.1), K70R/E (8, 6.3), M184V (31, 24.4), T215F/Y (10, 7.9), K219Q/E/R (7, 5.5), K65R (9, 7.1), K70Q (2, 1.6), Y115F (7, 5.5), A62V (3, 2.4), L74I (3, 2.4), M41L (5, 3.9), V75M/I (3, 2.4), E44A (1, 0.8), L210L/W (1, 0.8)
AZT	11	8.7	
D4T	25	19.7	
DDI	27	21.3	
FTC	36	28.3	
3TC	36	28.3	
TDF	22	17.3	
NNRTIs	46	36.2	
DOR	36	28.3	V179D/E (9, 7.1), Y181C (14, 11.0), K101E (8, 6.3), G190S/A/E (13, 10.2), K103N/S (20, 15.7), E138A (7, 5.5), H221Y (6, 4.7), M230L (3, 2.4), V106M/I (13, 10.2), F227L (2, 1.6), A98G (3, 2.4), P225H (3, 2.4), V108I (2, 1.6), Y188L (2, 1.6), L234I (2, 1.6)
EFV	45	35.4	
ETR	24	18.9	
NVP	45	35.4	
RPV	31	24.4	

PIs, protease inhibitors; NRTIs, nucleoside reverse transcriptase inhibitors; NNRTIs, non-nucleoside reverse transcriptase inhibitors. ATV, atazanavir; DRV, darunavir; FPV, fosamprenavir; IDV, indinavir; LPV/r, lopinavir/ritonavir; NFV, nelfinavir; SQV, saquinavir; TPV, tipranavir; ABC, abacavir; AZT, zidovudine; D4T, stavudine; DDI, didanosine; FTC, emtricitabine; 3TC, lamivudine; TDF, tenofovir disoproxil; DOR, dorivirine; EFV, efavirenz; ETR, etravirine; NVP, nevirapine; RPV, rilpivirine.

**Table 7:** Comparison of the number of newly reported infections linked to the failure of ART in different viral load groups in Hefei.

VL * (Copies/mL)	Total	Link to Newly Reported Infections	*Z*	*p*
1000–10,000	58	13 (21.7)	−2.98	0.001
10,000–50,000	41	21 (35.0)		
>50,000	28	26 (43.3)		

* VL, viral load.

## Data Availability

All data are contained within the article.
